# Therapeutic efficacy of chloroquine and primaquine for *Plasmodium vivax* malaria treatment in southeast Iran

**DOI:** 10.1186/1475-2875-11-S1-P45

**Published:** 2012-10-15

**Authors:** Aliehsan Heidari, Hossein Keshavarz, Ahmad Raeisi

**Affiliations:** 1 Dept. of Pathobiology, School of Medicine, Alborz University of Medical Sciences, Karaj, Iran; 2 Dept. of Medical Parasitology and Mycology, School of Public Health, Tehran University of Medical Sciences, lran; 3 Dept. of Medical Entomology and Vector Control, School of Public Health, Tehran University of Medical Sciences, Iran

## Background

*Plasmodium vivax* is the main cause of malaria infection in Asian, Central and South American countries [1]. It accounts for more than 90% annually of the reported malaria cases in Iran [2]. *Plasmodium vivax* resistant to chloroquine has emerged in some regions of Asia and resistance or tolerance to primaquine has been demonstrated in several countries [3,4]. The aim of this study was to determine the therapeutic efficacy of chloroquine and primaquine for *Plasmodium vivax* malaria treatment in southeast Iran.

## Material and methods

A total of randomly selected 270 patients with confirmed *P. vivax* infection participated in 28-day *in vivo* study that extended for 2 years for detecting relapse infection. Chloroquine and primaquine were administrated during 3 days and 8 weeks respectively in 2010. The thick and thin film blood smears were screened for malaria parasites by microscopy. The nested PCR was applied using the *Plasmodium* 18 subunit ribosomal ribonucleic (Ssr RNA) genes for detecting mixed infections and diagnosis of parasites in the samples with low parasite on days monitoring the drug resistance.

## Results

Fever resolved on the first day in all subjects. Microscopy findings showed that *P. vivax* was cleared in 15%, 50%, 95%, and 100% of patients on days 1, 2, 3 and 4, respectively. All 270 subjects showed ~120 Bp band in the nested PCR which was indicative of P. *vivax* malaria on the zero days. Six patients (2.2%) had specific *P. vivax* band in nested PCR on day 5. No recurrence was observed on days 7, 14 and 28 in thick blood smear and nested PCR. Mean (±standard deviation) parasite clearance time was 2.41 (±0.8) days. Two patients had *P. vivax* malaria clinical and parasitological infection following 8 and 12 months after primary *P. vivax* malaria infection.

## Conclusions

The findings of this study showed susceptibility of P. *vivax* to chloroquine in South east Iran. This finding is compatible with results of neighboring countries Pakistan and Afghanistan. Nested PCR was a suitable assay to determine exact malaria parasite clearance time in our study. The further investigation is being conducted in two reinfection cases by PCR -Single strand conformational polymorphism method to differentiate between relapse and new P. *vivax* infection.
